# A Bifunctional Organic Redox Catalyst for Rechargeable Lithium–Oxygen Batteries with Enhanced Performances

**DOI:** 10.1002/advs.201500285

**Published:** 2015-12-16

**Authors:** Jinqiang Zhang, Bing Sun, Xiuqiang Xie, Yufei Zhao, Guoxiu Wang

**Affiliations:** ^1^ Centre for Clean Energy TechnologySchool of Mathematical and Physical SciencesUniversity of Technology SydneyNSW2007Australia

**Keywords:** bifunctionality, lithium–oxygen battery, organic catalysts, PTMA

## Abstract

**An organic bifunctional catalyst** poly(2,2,6,6‐tetramethylpiperidinyloxy‐4‐yl methacrylate) (PTMA) has been prepared and coated on carbon surface during electrode preparation. The PTMA has been applied as an efficient bifunctional catalyst for lithium–oxygen batteries with lower overpotentials, enhanced rate performances, and prolonged cycle life.

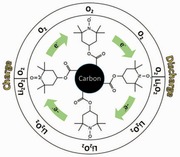

The lithium–oxygen (Li–O_2_) battery has attracted considerable interest owing to its high energy density.^1^, ^2^, ^3^, ^4^, ^5^ Some critical drawbacks of Li–O_2_ batteries have, however, limited its practical application to date. For instance, the formation of superoxide radical species (O_2_^.−^) causes serious problems.^6^, ^7^ Due to its highly reactive nature, O_2_^.−^ can attack the electrolyte and electrode materials when Li_2_O_2_ (the ideal oxygen reduction product) is formed resulting in large amounts of unwanted byproducts. In efforts to address this, the use of ionic liquids as electrolytes and porous gold electrodes has been investigated.^3^, ^8^, ^9^ These materials achieve long and stable cycle lives. However, these materials are usually very expensive and difficult to prepare. Additionally, large overpotentials during charge and discharge processes, resulting from the sluggish kinetics of the battery reaction, lead to poor round‐trip efficiency and cycle life.^10^ Therefore, catalysts have been utilized to reduce the overpotentials and to increase the cycle life. Potential catalysts such as metals, metal oxides, perovskite, carbon nanotubes, graphene, and organic compounds have been investigated.^10^, ^11^, ^12^, ^13^, ^14^, ^15^, ^16^, ^17^, ^18^, ^19^, ^20^, ^21^, ^22^, ^23^, ^24^ Ruthenium metal has recently demonstrated superior capability to reduce charge overpotential during the oxygen evolution reaction (OER) over other catalysts.^25^ Furthermore, redox mediators such as tetrathiafulvalene, 2,2,6,6‐tetramethyl‐1‐piperidinyloxy (TEMPO), lithium iodide, iron phthalocyanine, and methyl‐10H‐phenothiazine have shown the capability to enhance the round‐trip efficiency and suppress side reactions by reducing the charge potential to below 4.0 V.^26^, ^27^, ^28^, ^29^, ^30^, ^31^, ^32^ From an overall perspective, and because the oxygen reduction reaction (ORR) also plays an important role, it is desirable to develop catalysts with bifunctionality for both discharge and charge processes. For example, by mixing highly efficient OER catalysts with gold, which has good catalytic activity toward the ORR, the gap between discharge and charge voltage can be significantly reduced.^33^ Noble metal catalysts are not ideal owing to their high cost, and therefore, it is crucial to discover low cost catalysts with high catalytic activity. Recently, Zhu et al. reported the use of a combination of two organic redox mediators, in which one catalyzes the ORR and the other catalyzes the OER.^34^

Herein, we report the remarkable properties of poly(2,2,6,6‐tetramethylpiperidinyloxy‐4‐yl methacrylate) (PTMA) as an organic catalyst in Li–O_2_ batteries. During the discharge process, PTMA in its n‐doping state catalyzes O_2_ reduction and formation of Li_2_O_2_, while during the charge process, PTMA converts to its p‐doping form and facilitates Li_2_O_2_ decomposition. Furthermore, PTMA forms a protective layer to suppress side reactions between the carbon electrode and the electrolyte.

PTMA was synthesized using the previously reported method (Figure S1a, Supporting Information)^35^, ^36^ and has a distinctive red color (Figure S1b, Supporting Information). The Fourier transform infrared spectroscopy (FTIR) spectra confirmed that PTMA had been successfully synthesized (Figure S2, Supporting Information). PTMA electrodes were prepared by mechanically grinding PTMA and carbon black (CB) with the binder polyvinylidene difluoride. The mixture was dispersed in N‐methyl‐2‐pyrrolidone (NMP) and then cast to form electrodes. The PTMA was found to uniformly distribute and coat onto the surface of carbon black (**Figure** **1** and Figure S3, Supporting Information) due to its good solubility in NMP (Figure S4, Supporting Information). Scanning electron microscopy (SEM) mapping images of the electrode (Figure S5, Supporting Information) confirm an even coating of PTMA on the surface of CB. FTIR spectra of PTMA and CB before and after dissolving in NMP are shown in Figure S6 (Supporting Information), which confirms that the dissolving process did not affect the structure of PTMA. Figure S7a (Supporting Information) demonstrated that PTMA is not soluble in diethylene glycol dimethyl ether (DEGDME), which is consistent with the previous report.^37^ Furthermore, we measured the FTIR spectra of DEGDME solvent and DEGDME soaked with PTMA for 2 h. As shown in Figure S7b (Supporting Information), there is no additional peak appeared in the FTIR spectra of DEGDME solvent after soaked with PTMA. This result clearly verified the insolubility of PTMA in the DEGDME solvent.

**Figure 1:**
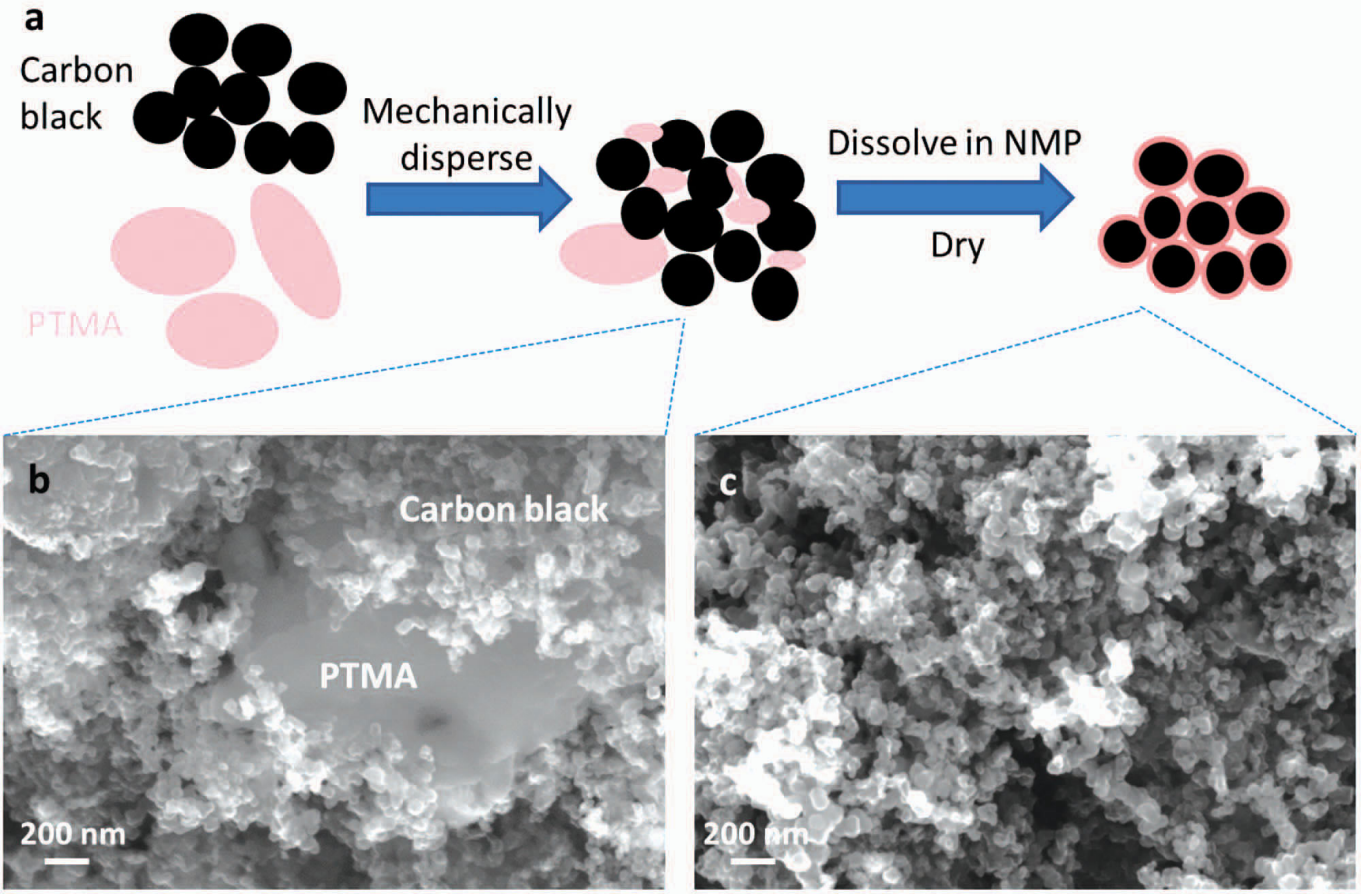
a) Illustration of the processes to form the PTMA/carbon black electrode material. Insets show SEM images of the materials b) before and c) after dissolving in NMP.

Li–O_2_ cells were constructed using the new PTMA electrodes as cathode and lithium metal as the counter electrode and reference electrode. Cells prepared with bare CB electrodes were also tested for comparison. **Figure** **2** shows the cyclic voltammetry (CV) curves measured from 2 to 4.5 V with a scanning rate of 0.1 mV s^−1^. No significant redox processes were observed for the bare CB electrode. In contrast, a process at ≈3.8 V is evident in the cell containing the PTMA electrode, which is related to the p‐doping of PTMA. Importantly, the potential of this process meets the essential requirement of decomposing Li_2_O_2_. An additional redox process was identified at 2.96 V, which is the theoretical potential of the formation of Li_2_O_2_.^1^, ^2^, ^3^, ^4^ We assign this process to n‐doping of PTMA.^35^, ^38^ Cell discharge usually occurs at ≈2.7 V and it is important that the catalyst has a slightly more positive redox potential to allow the optimum battery discharge behavior. These data show that PTMA can catalyze the decomposition of Li_2_O_2_ (upon charging) and also facilitate the formation of Li_2_O_2_ (upon discharging). The CV curve of Li–O_2_ battery with PTMA electrode in O_2_ atmosphere is shown in Figure S8 (Supporting Information), which also confirms the catalytic activity of PTMA toward ORR and OER.

**Figure 2:**
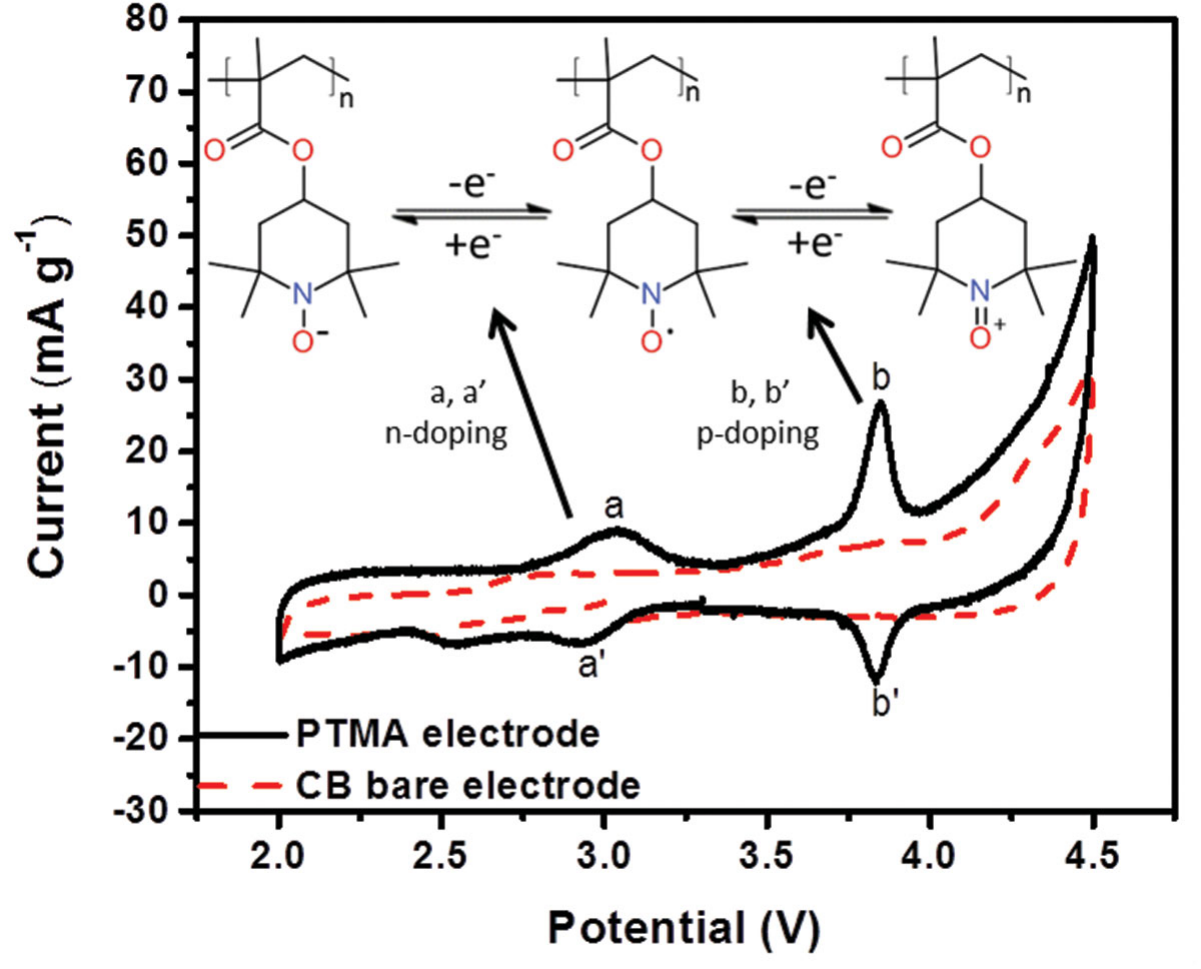
The cyclic voltammetry curves of the sealed cells in an argon atmosphere with PTMA and bare CB electrodes. Scanning rate is 0.1 mV s^−1^ and potential range is 2.0–4.5 V. The inset is the redox reactions of PTMA during p‐doping and n‐doping.

Galvanostatic charge/discharge testing was conducted to evaluate the electrochemical performance of the Li–O_2_ batteries under a restricted capacity of 1000 mAh g^−1^. The results are shown in **Figure** **3**a. The charge plateau of the Li–O_2_ battery with a PTMA electrode presents a voltage of about 3.73 V, which is significantly lower than that of the bare CB electrode (4.15 V). This result indicates that PTMA can reduce the charge overpotentials during OER. The difference between the discharge plateaus of the PTMA and CB electrodes is about 0.06 V, which reveals the superior catalytic activity of PTMA toward ORR over CB (see also d*Q*/d*V* vs potential graph of the discharge–charge processes, Figure S9, Supporting Information). The fully discharge/charge profiles were obtained with a cut‐off voltage at 2.3 V/4.5 V (shown in Figure S10, Supporting Information). The results clearly indicate that PTMA can not only reduce the overpotentials during discharge and charge processes but also increase discharge capacity, comparing with bare CB electrode. The discharge/charge profile of cell with PTMA electrode in argon atmosphere (Figure S11, Supporting Information) also confirms that the discharge capacity originates from the catalyzed ORR rather than PTMA itself.

**Figure 3:**
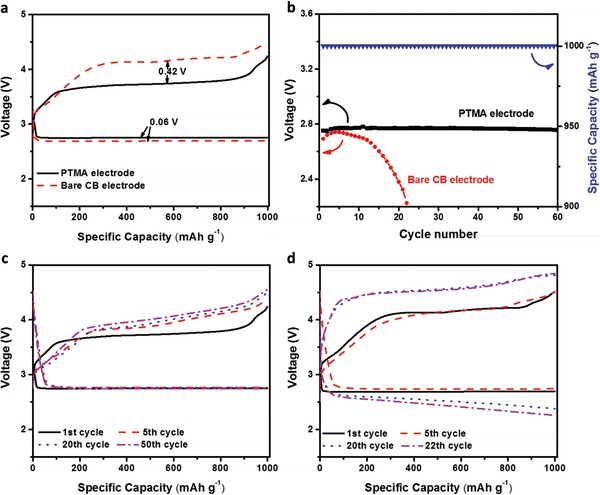
a) Discharge/charge profiles of Li–O_2_ batteries with PTMA electrode and bare CB electrode at a discharge depth of 1000 mAh g^−1^ and a current density of 200 mA g^−1^. b) Cycling profile of both Li–O_2_ batteries. And discharge/charge profile during cycling of Li–O_2_ batteries with c) PTMA electrode and d) bare CB electrode. The cut‐off voltage was set to be 2.3 V/4.8 V. The current density and capacities were calculated by the weight of the active materials in the electrodes (PTMA + CB and CB).

Cycling performances of PTMA electrode in Li–O_2_ batteries were evaluated by constant current discharge/charge cycling (shown in Figure 3b–d). The discharge and charge capacities were fixed to 1000 mAh g^−1^, and the current density was 200 mA g^−1^. The current density and capacities were calculated based on the weight of active materials in the electrodes (PTMA + CB and CB). Without PTMA in the electrode, the overpotentials of Li–O_2_ battery continuously increased with cycling until terminating at the 23rd cycle (cut‐off voltage was 2.3 V). However, with the addition of PTMA, the Li–O_2_ battery lasted at least 60 cycles without any degradation. The discharge curves exhibited a constant flat plateau at around 2.75 V, whereas the plateaus of charge slightly increased with cycling, but remained lower than 4 V even at the 50th cycle. In general, the termination of Li–O_2_ batteries is usually caused by the decomposition of the electrolyte or binder and the formation of side products at voltages higher than 4 V.^39^ As shown in Figure 3a, the PTMA catalyst can efficiently reduce the overpotential during the charge process, which can suppress the side reactions and therefore improve cycling performance.

Li–O_2_ batteries with PTMA electrodes were assembled and cycled at different current densities (Figure S12, Supporting Information). The current densities do not have a significant impact on the discharge–charge performance under a restricted capacity of 1000 mAh g^−1^. The voltages of the charge plateaus only slightly increased when the current densities increased from 50 to 1000 mAh g^−1^. When using bare CB electrode, the overpotentials in both discharge and charge processes increased dramatically (as shown in Figure S12, Supporting Information). The fully discharge and charge profiles in Figure S13 (Supporting Information) also confirm good rate capability of the catalysts. We ascribe the significantly enhanced performances of the PTMA electrodes to the fast kinetics of the PTMA redox reactions. Thus, the PTMA electrode has a demonstrated high rate capability.

SEM and X‐ray diffraction (XRD) were used to characterize PTMA electrodes after discharge and charge cycles. SEM images (Figure S14b, Supporting Information) show the formation of nanosized Li_2_O_2_ after discharge and large amounts of Li_2_O_2_ can be detected on the surface of the cathode. High magnification images (Figure S14b (inset), Supporting Information) show Li_2_O_2_ plates with a toroidal shape, which is consistent with previous reports.^3^, ^10^, ^40^ The XRD results (Figure S14d, Supporting Information) confirm that the products were dominated by Li_2_O_2_. The FTIR spectrum in Figure S15 (Supporting Information) further verified this conclusion. After charging, the toroidal‐shaped Li_2_O_2_ disappeared (Figure S14c, Supporting Information), indicating the reversibility of Li_2_O_2_ formation and decomposition.

We propose that PTMA functions as a bifunctional organic redox catalyst in Li–O_2_ batteries. The nature of n‐/p‐doping of PTMA during discharge and charge processes contributes to the significant decrease of overpotentials. According to previous reports, PTMA or materials containing the TEMPO moieties can work as a catalyst for oxidation and reduction reactions by directly interacting with the reactants, in addition to acting as a redox electron mediator.^41^, ^42^ The result of standard catalytic activities toward ORR and OER (Figure S16, Supporting Information) also indicates that PTMA has a rapid and efficient catalytic capability.

**Figure** **4** illustrates the proposed mechanisms of the PTMA‐catalyzed formation and decomposition of Li_2_O_2_ in the Li–O_2_ battery. For clarity, the structure of PTMA is replaced by the main functional group, the nitride oxide radical. Figure 4a shows the discharge process whereby the nitride oxide radical moiety is firstly reduced to the n‐doped form, and a Li^+^ is drawn from electrolyte to balance the charge. Due to the attraction between Li^+^ and oxygen, the oxygen molecules were absorbed to the surface of the PTMA electrode. By interacting with the reduced nitride oxide moiety through Li^+^, oxygen is then reduced to Li_2_O_2_ while the n‐doped nitride oxide moiety reverts to its radical state. This catalytic cycle proceeds to produce more Li_2_O_2_ product. The catalytic effect of PTMA plays an important role in facilitating the formation of Li_2_O_2_ as well as suppressing the formation of side products. Moreover, the similar radical nature of PTMA could make it much easier to attract and reduce the superoxide radicals (O_2_^.−^) formed during the discharge process, which also reduces the risk of electrolyte decomposition.^43^

**Figure 4:**
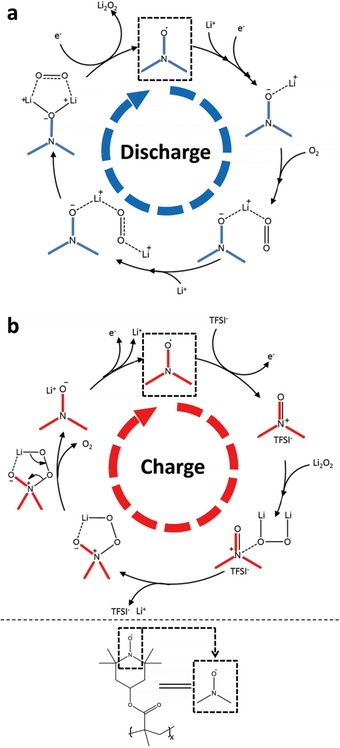
Schematic illustration of mechanism of PTMA during discharge and charge processes. To simplify the illustration, the structure of PTMA is replaced by its nitride oxide moiety as it is the main functional group.

A similar mechanism is proposed for the charge process (Figure 4b). The oxidized nitride oxide moiety interacts with Li_2_O_2_ and releases one Li^+^. The other Li^+^ was attracted by the oxygen atom from the oxidized nitride oxide moiety. By rearranging the electrons in the intermediate structure, the oxygen was then released. Meanwhile, the other Li^+^ remains attached to the PTMA surface. With the flow of electrons from the electrode, PTMA reverts back into its original radical form. This process reduces the reaction energy barrier for Li_2_O_2_ decomposition, which results in the decrease of charge overpotential. In addition, a lower charge voltage may suppress the decomposition of carbon and electrolyte. Moreover, the PTMA coating can prevent direct contact between Li_2_O_2_ and the carbon surface, reducing side reactions between them.

The use of catalysts in Li–O_2_ batteries has been intensively debated recently with suggestions that catalysts could catalyze the decomposition of the electrolyte instead of Li_2_O_2_.^4^ It is therefore important to clarify whether PTMA has a superior catalytic activity toward Li_2_O_2_ oxidation than the electrolyte. The catalytic activities of PTMA toward the decomposition of Li_2_O_2_ were confirmed by linear sweep voltammetry, scanning from open circuit voltage to 4.4 V with a scanning rate of 0.1 mV s^−1^ (**Figure** **5**). Electrodes were prepared with or without the addition of commercial Li_2_O_2_. For the CB–Li_2_O_2_ electrode, the oxidation peak starting from 4.0 V originates evidently from the decomposition of Li_2_O_2_. For bare CB electrode there is no visible peak at the same potential range. A small peak can be identified above 4.3 V, which could be ascribed to the side reactions such as electrolyte decomposition. The bare PTMA electrode without the commercial Li_2_O_2_ shows a small additional peak at around 3.8 V, which is associated with the p‐doping of PTMA. There is no other oxidation peak observed for the bare PTMA electrode, indicating that negligible electrolyte decomposition at lower voltage range of 4.2 V. The PTMA–Li_2_O_2_ electrode exhibits a broad peak starting from 3.6 V, which can be assigned to the decomposition of Li_2_O_2_ under the catalysis of PTMA. The decomposition voltage is very close to the voltage of p‐doping of PTMA. This confirmed that PTMA has a strong catalytic activity toward the decomposition of Li_2_O_2_.

**Figure 5:**
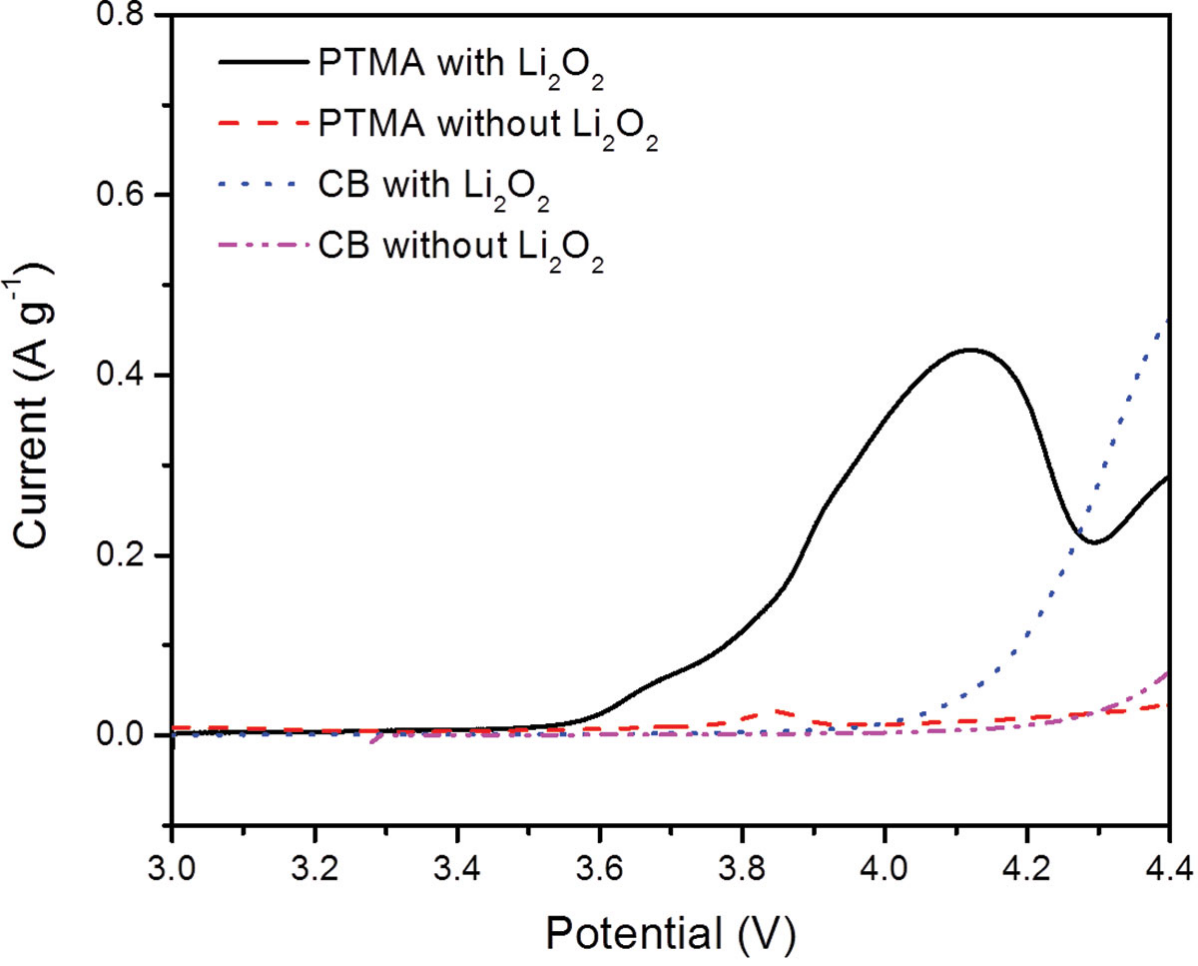
Liner sweep voltammetry of electrode with and without commercial Li_2_O_2_. The scanning rate is 0.1 mV s^−1^. The range is set from open circuit voltage to 4.4 V.

In summary, a reversible Li–O_2_ battery with high efficiency was demonstrated using a bifunctional organic catalyst, PTMA. This material can lower the overpotentials both in discharge and in charge processes due to its redox doping nature and catalytic properties. The coating of this efficient catalyst onto the carbon surface also reduced side reactions between carbon and electrolyte, leading to a prolonged cycle life.

## Supporting information

As a service to our authors and readers, this journal provides supporting information supplied by the authors. Such materials are peer reviewed and may be re‐organized for online delivery, but are not copy‐edited or typeset. Technical support issues arising from supporting information (other than missing files) should be addressed to the authors.

Supplementary
