# Supplementary material for: A Bifunctional Organic Redox Catalyst for Rechargeable Lithium–Oxygen Batteries with Enhanced Performances
Source: Adv Sci (Weinh). 2015 Dec 16;3(4):1500285. doi: 10.1002/advs.201500285 (PMC5064662; doi:10.1002/advs.201500285)
Supplement: Supplementary file 1 — Supplementary [file ADVS-3-0n-s001.pdf]

Copyright WILEY-VCH Verlag GmbH & Co. KGaA, 69469 Weinheim, Germany, 2015.

ADVANCED SCIENCE
Open Access

Supporting Information

for *Adv. Sci.*, DOI: 10.1002/advs. 201500285

A Bifunctional Organic Redox Catalyst for Rechargeable Lithium–Oxygen Batteries with Enhanced Performances

*Jinqiang Zhang, Bing Sun, Xiuqiang Xie, Yufei Zhao, and Guoxiu Wang**

Copyright WILEY-VCH Verlag GmbH & Co. KGaA, 69469 Weinheim, Germany, 2013.

Supporting Information

A Bi-functional Organic Redox Catalyst for Rechargeable Lithium-Oxygen Batteries with Enhanced Performances

*Jinqiang Zhang, Bing Sun, Xiuqiang Xie, Yufei Zhao, Guoxiu Wang**

Experimental section

Synthesis of PTMA

PTMA was synthesized according to the previously reported method.[1,2] In a typical synthesis process, 2.25 g 2,2,6,6-tetramethyl-4-piperidy methacrylate (MTMP, TCI) monomer and 0.04 g 2,2'-azobisisobutyronitrile (AIBN, Sigma-Aldrich) were mixed and stirred in 6 mL acetic acid at 70 °C for 12 h under Ar atmosphere. The mixture was then added into 50 mL ethyl ether (Chem-Supply). The pale white product (PMTMP) (1.7 g, 75 % yield) was obtained by filtration.

The product PMTMP (1.0 g) was then dissolved into 20 mL methanol (Chem-Supply), mixed with 0.3 g $Na_2WO_4 \cdot 2H_2O$ (Sigma-Aldrich), 0.2 g ethylenediaminetetraacetic acid (EDTA, Sigma-Aldrich), 2 mL 30 % H_2O_2 (TCI), and 10 mL H_2O. The mixture was then stirred at 60 °C for 40 h. The product was filtrated and washed with de-ionised water and ethyl ether (Chem-Supply) to obtain PTMA (a pale red solid (1.0 g, 95 % yield)).

The illustration of the process is shown in Figure S1a.

Characterization

Field emission scanning electron microscope (FESEM, Zeiss Supra 55 VP) was employed to observe the morphologies. Infrared spectroscopies of MTMP and the as-prepared PTMA were conducted on a Nicolet Magna 6700 FT-IR spectrometer. All spectra were obtained using 4 cm^{-1} resolution and 64 scans at room temperature. X-ray diffraction (XRD) measurement was conducted on a Siemens D5000 X-ray diffractionmeter. During the XRD analysis process, the cathodes were protected from exposure to the ambient atmosphere.

Electrochemical characterization

The cathodes were prepared by firstly mixing carbon black, PTMA, and polyvinylidene difluoride (PVDF) together in methylpyrrolidone (NMP, Sigma-Aldrich) with the weight ratio of 60:30:10. The mixture was then coated on a stainless steel mesh substrate and cut into discs. The electrodes were dried at 80 ºC in a vacuum oven for 24 h. The loading of the whole cathode materials (PTMA+CB+binder PVDF) is 1 mg cm^{-2}. The carbon black electrodes were prepared by the same process without the addition of PTMA. The Li_2O_2 electrodes were prepared with the addition of commercial Li_2O_2 (weight ratio of Li_2O_2, active materials PTMA+CB or CB, and binder PVDF is 1:4:1).

All the electrochemical characterizations were conducted on a CH Instrument 660D electrochemical workstation and discharge/charge performances were evaluated by a Neware Battery Testing System. The cyclic voltammetry measurements were carried on within the range from 2 V to 4.5 V, with a scanning rate of 0.1 mV s^{-1}. Linear sweep voltammetry measurements were performed to measure the catalytic property of materials towards the decomposition of Li_2O_2. The scanning rate was 0.1 mV s^{-1} and the range was set from open circuit voltage to 4.4 V.

The discharge-charge performances were evaluated by assembling Li-O_2 batteries. A two-electrode system Swagelok-type cell with an air hole (0.785 cm^2) on the cathode side was used to test the electrochemical performances. The cell was assembled in an argon filled glove box with water and oxygen level less than 0.1 ppm. A lithium foil was used as anode. The electrolyte was prepared by dissolving bis(trifluoromethane) sulfonimide lithium salt (LiTFSI, Sigma-Aldrich, 99.95 %) in diethylene glycol dimethyl ether (DEGDME, Sigma-Aldrich, 99 %, anhydrous). The solvent was pre-dried by molecular sieves (4A, Sigma-Aldrich) for at least one week before use. The concentration was 0.5 M. The assembled cell was gas tight except for the cathode side window, which is exposed to the oxygen atmosphere. All measurements were conducted in 1 atm dry oxygen atmosphere. The capacities of the batteries were calculated based on the total mass of the active materials (excluding binder). The mass of active materials is around 0.707 mg for each electrode.

The standard oxygen evolution reaction (OER) and oxygen reduction reaction (ORR) were performed in O_2–saturated 1 M KOH aqueous solution using a three electrode system on an electrochemical workstation (CHI 660E). The platinum wire and Ag/AgCl (1 M KCl) work as the counter and reference electrodes ($E_{RHE} = E_{Ag/AgCl} + 0.059\times pH + 0.2224$), respectively. The working electrode was prepared by loading a catalyst layer on the glass carbon substrate of rotating disk electrode (RDE, Pine) with a diameter of 5 mm. 4 mg of catalyst and 80 µl of Nafion (5 wt %) were mixed in 1 ml solvent (1:1 v/v water/isopropanol) by sonication for more than 30 min to obtain a

homogeneous ink. Then, the catalyst ink (10 µl) was loaded onto RDE followed by drying at room temperature. The disk electrode was scanned at a rate of 5 mV s^{-1} for OER and ORR. During the OER measurement, the working electrode was rotated at 1600 rpm to remove the generated oxygen bubbles. And during the ORR measurement, the working electrode was scanned with varying rotating speeds from 400 rpm to 2000 rpm.

[1] W. Guo, Y.-X. Yin, S. Xin, Y.-G. Guo, L.-J. Wan, *Energy Environ. Sci.*, **2012**, *5*, 5221-5225.

[2] J.-K. Kim, J.-H. Ahn, G. Cheruvally, G.S. Chauhan, J.-W. Choi, D.-S. Kim, H.-J. Ahn, S.H. Lee, C.E. Song, *Met. Mater. Int.*, **2009**, *15*, 77-82.

Supporting Figures

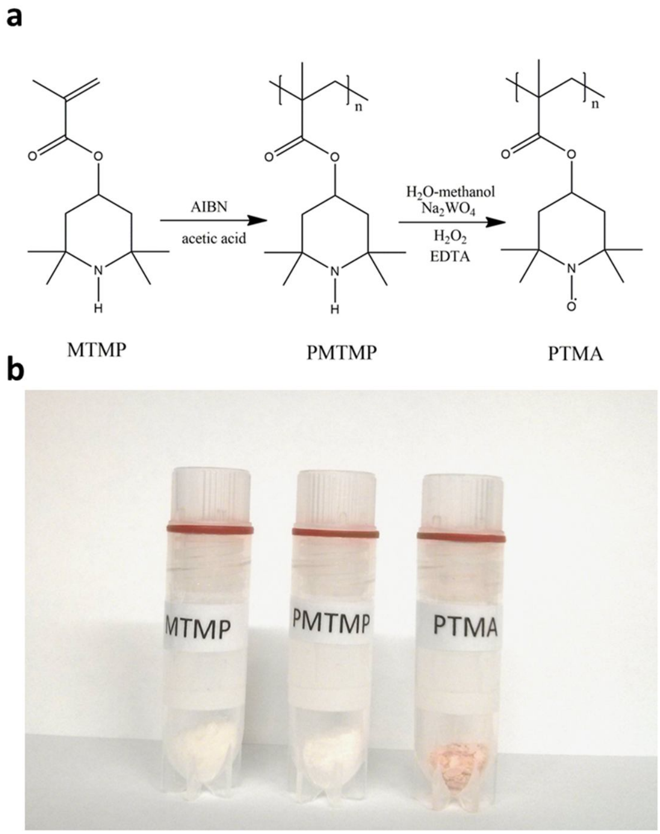

Figure S1. (a) The illustration of the PTMA synthesis process and (b) digital photo of the as-prepared PTMA, reactant MTMP, and intermediate PMTMP.

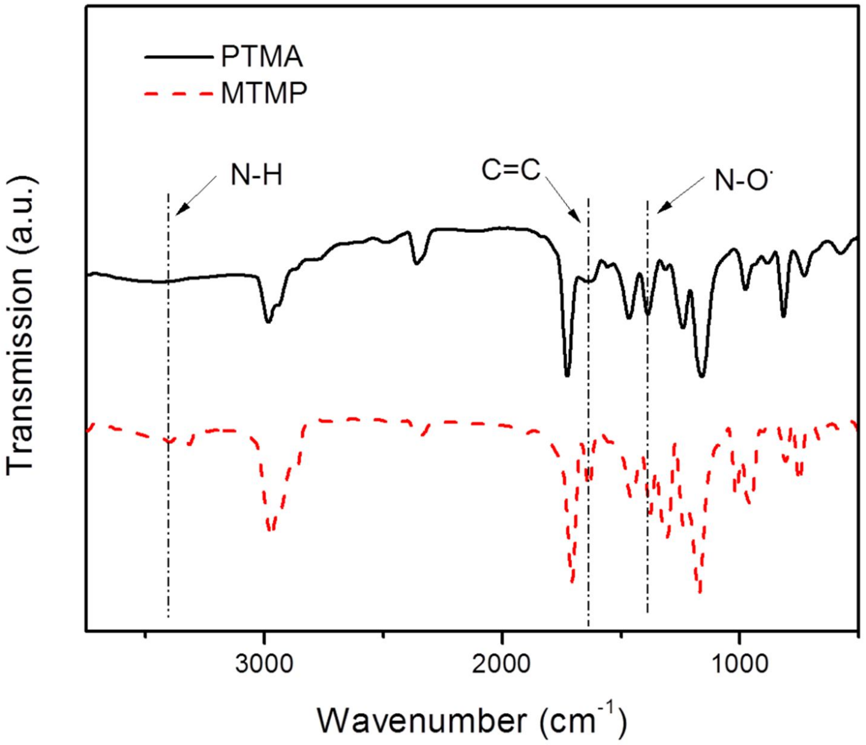

Figure S2. FT-IR spectra of the as-prepared MTMP and PTMA. The characteristic peaks at 1635 cm^{-1} (C=C group), 1707 cm^{-1} (C=O group) are known to be assigned to the MTMP monomer. The broad peaks near 3400 cm^{-1} are from the group of N-H. From the spectra of PTMA, it is evident that the peaks originated from C=C and N-H has disappeared or partially disappeared. Instead a clear new peak at 1370 cm^{-1} appeared, corresponding to the formation of the nitroxyl radical of PTMA.

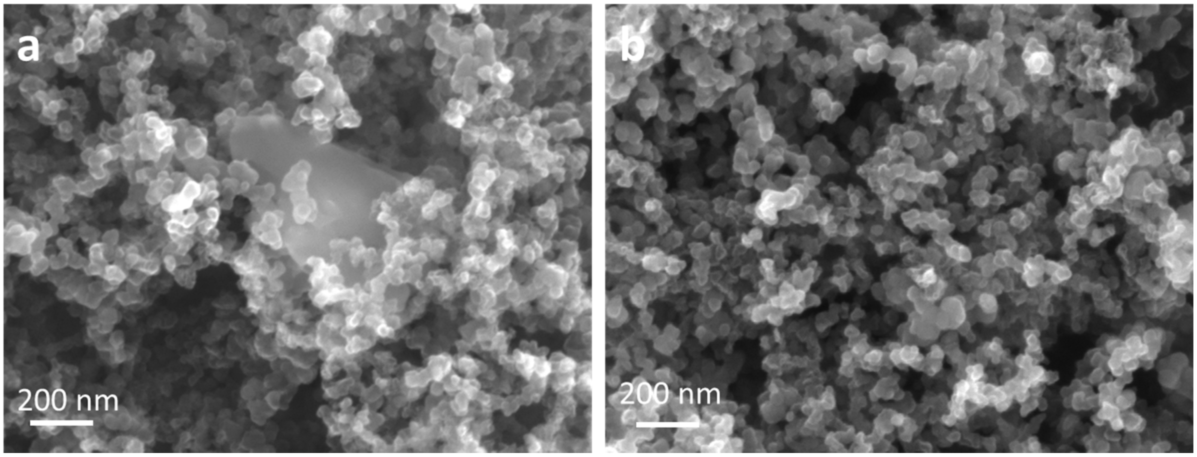

Figure S3. The SEM images of PTMA and carbon black mixture, (a) before and (b) after dissolving in NMP.

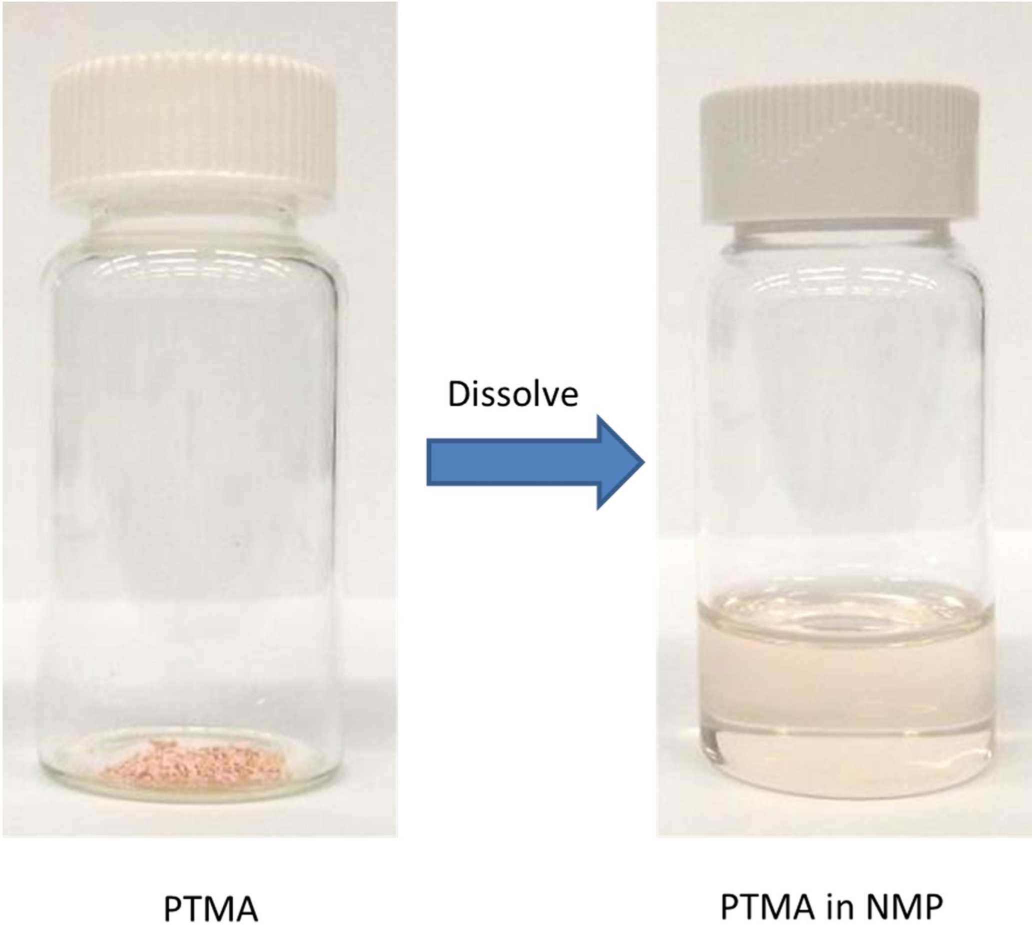

Figure S4. The digital photos of PTMA dissolving in NMP.

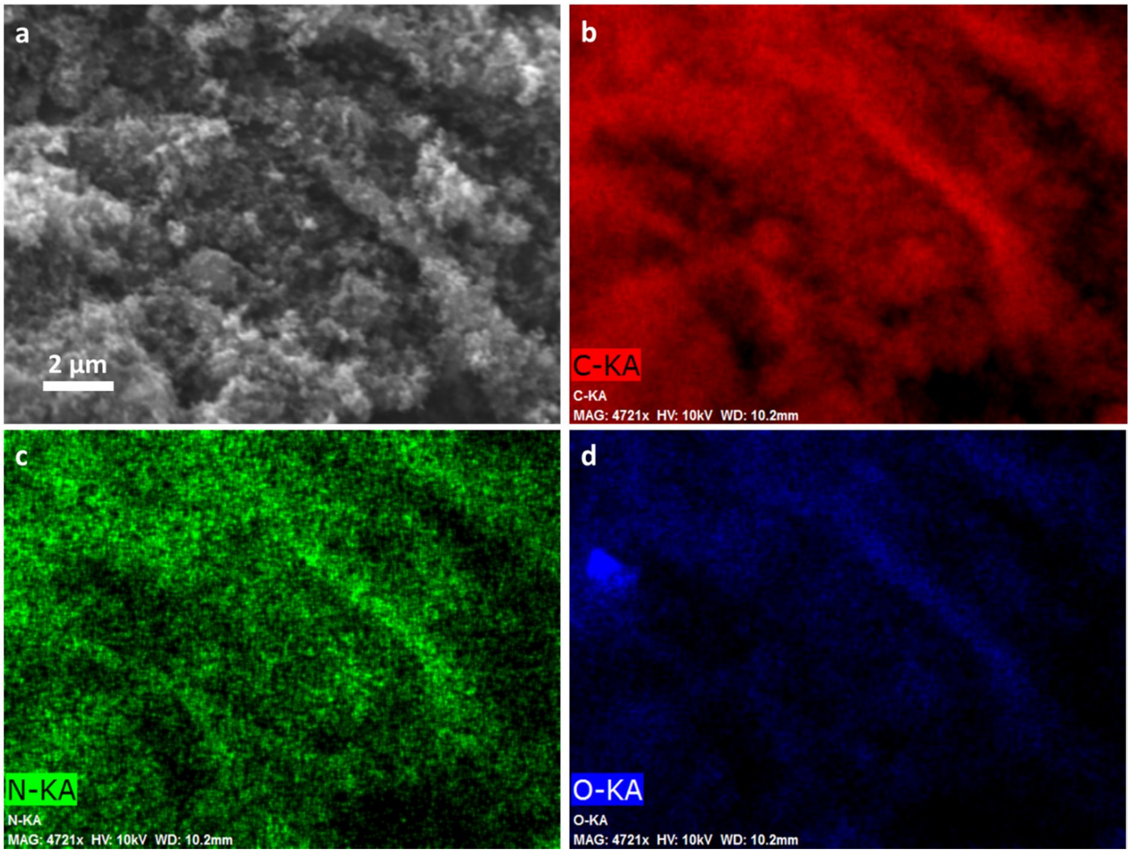

Figure S5. EDX mapping of the PTMA electrode.

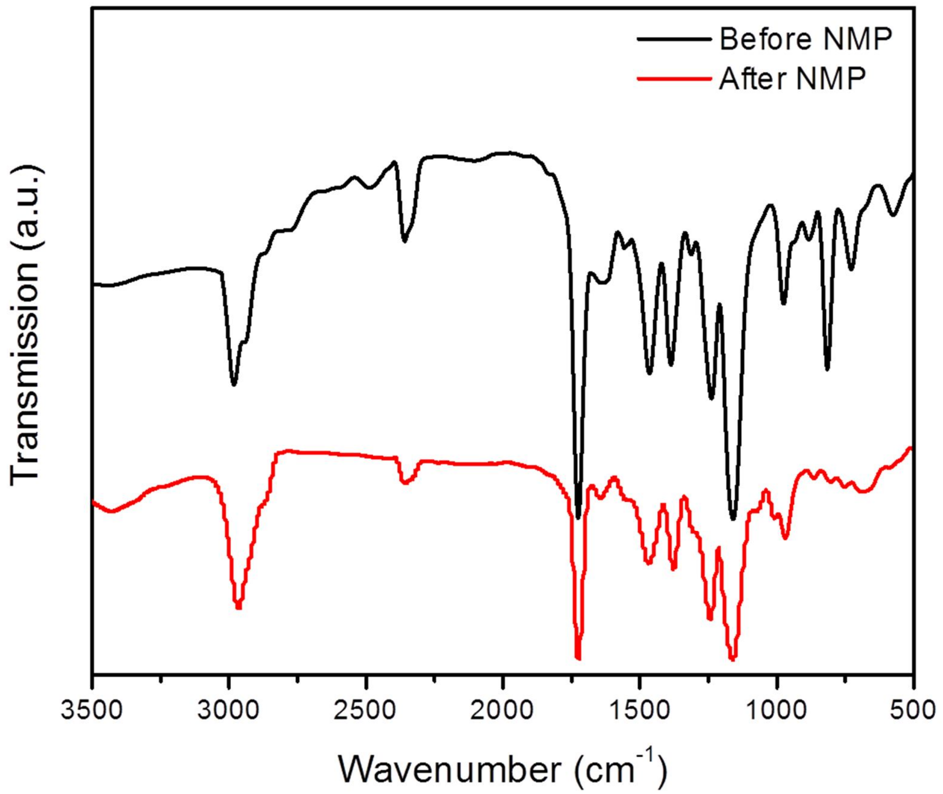

Figure S6. FTIR spectra of PTMA before and after dissolved in NMP.

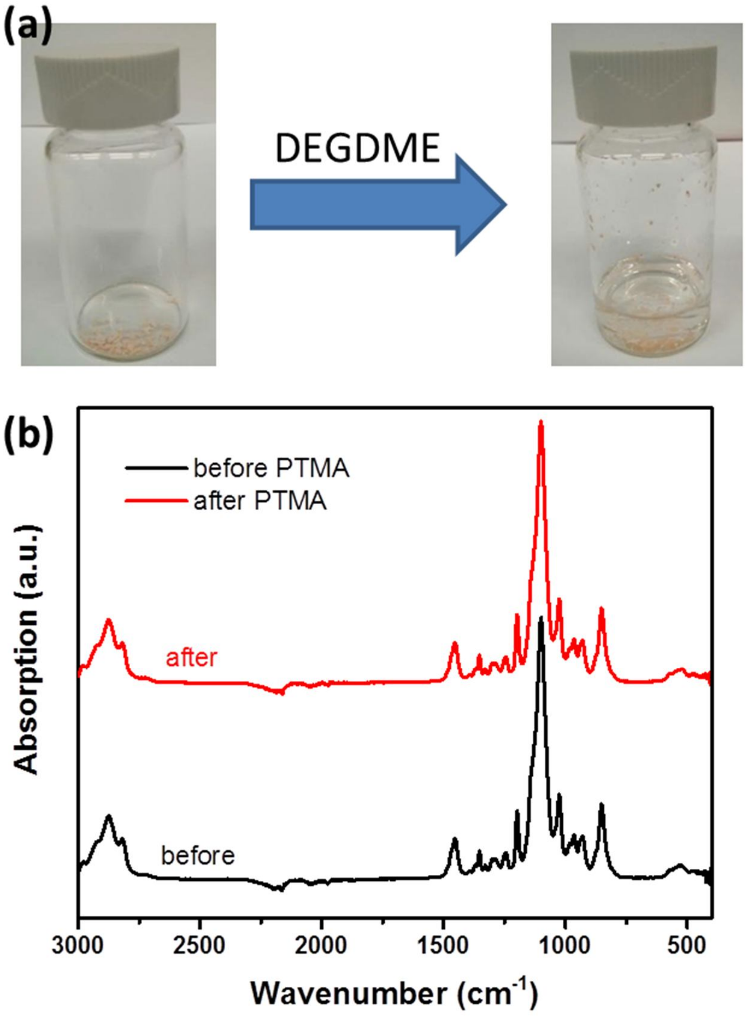

Figure S7. (a) Digital photos of PTMA before and after soaked in DEGDME solvent. (b) FTIR spectra of the DEGDME solvent before and after PTMA soaked for two hours.

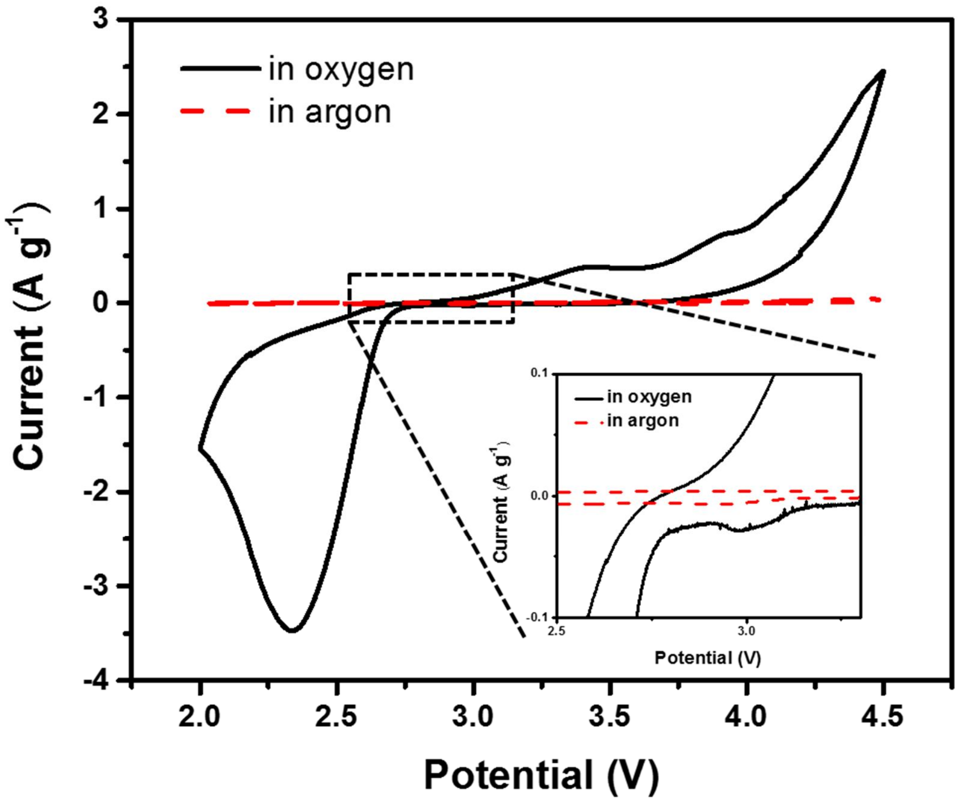

Figure S8. The CV curves of PTMA electrodes both in argon and oxygen atmosphere. Scanning rate is 0.1 mV s^{-1} and potential range is 2.0 to 4.5 V. The inset is the enlarged curves from 2.5 V to 3.3 V.

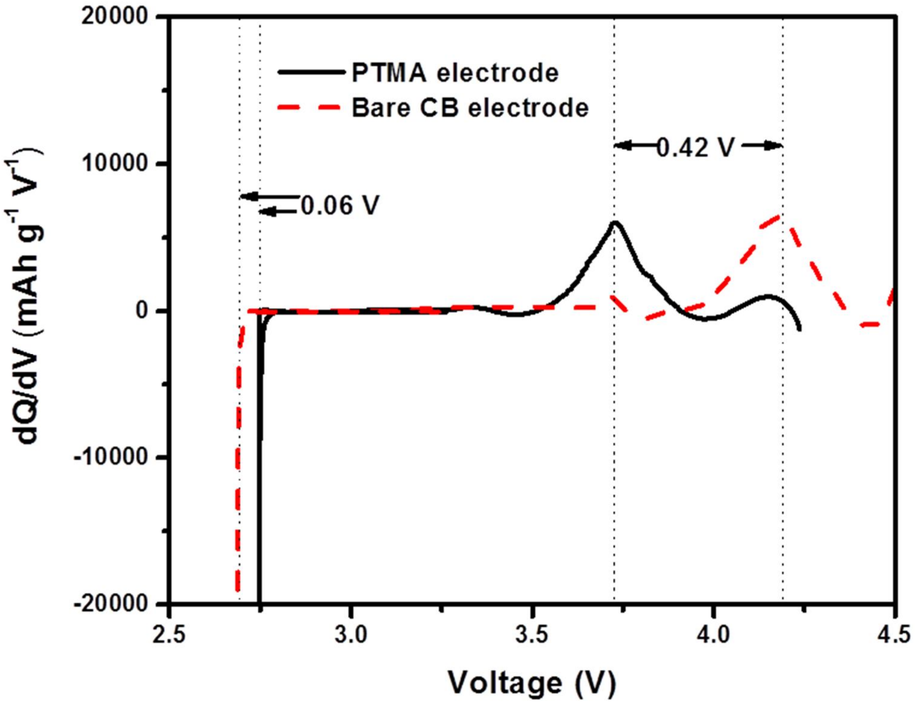

Figure S9. The dQ/dV *vs.* Voltage of Li-O_2 batteries during the first cycle under a restricted capacity of 1000 mAh g^{-1}.

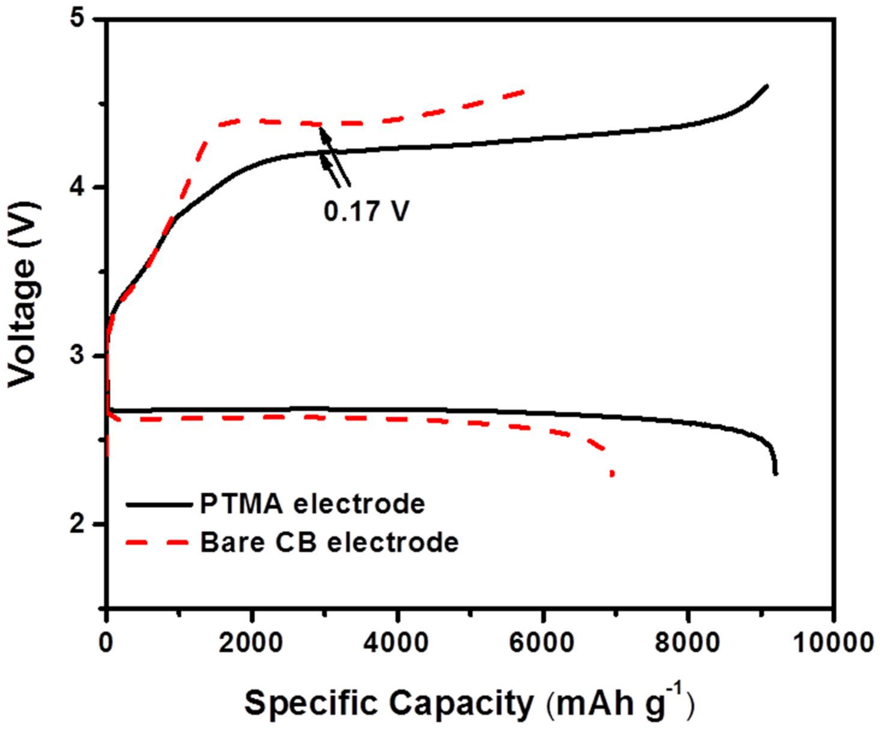

Figure S10. Fully discharge/charge profile of Li-O_2 batteries with PTMA and bare CB electrodes. The current density is 200 mAh g^{-1}. The cut-off voltage is set at 2.3 V/4.5 V.

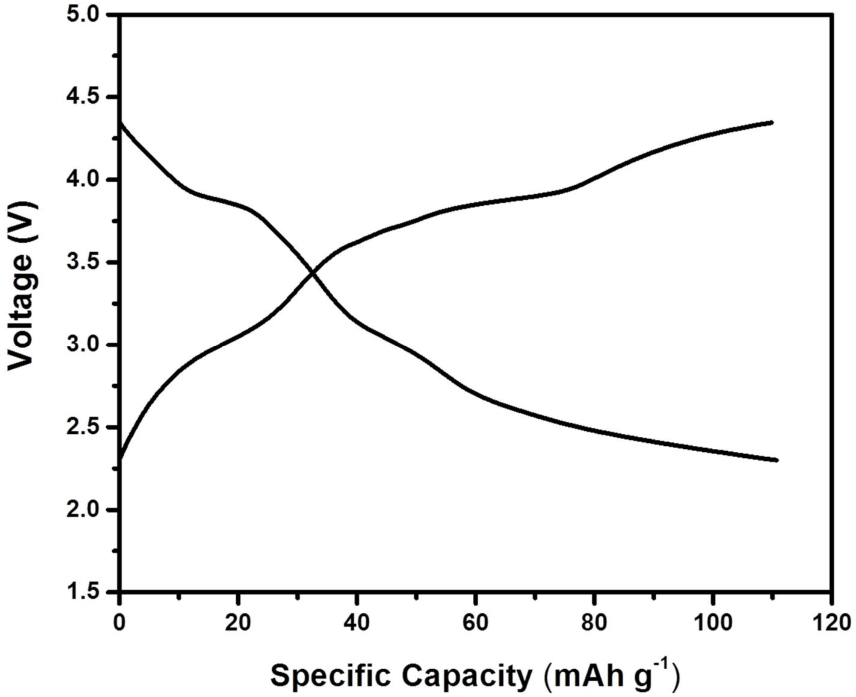

Figure S11. The full discharge/charge profile of the cell with PTMA electrode in Argon atmosphere. The current density is 200 mAh g^{-1}. The cut-off voltage is set as 2.3 V - 4.5 V.

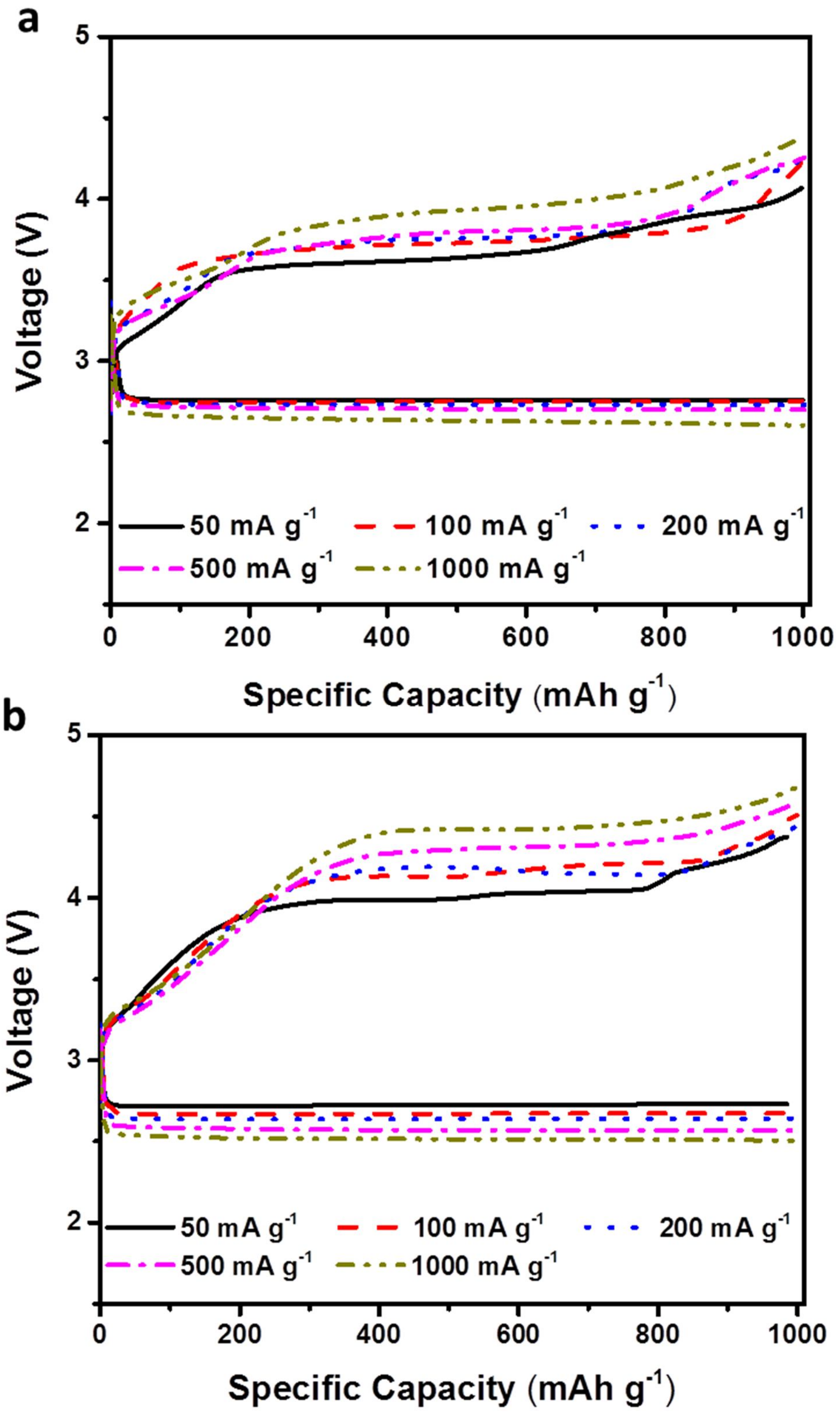

Figure S12. Rate capabilities of Li-O_2 batteries with PTMA (a), and bare CB (b) with current densities from 50 to 1000 mA g^{-1}.

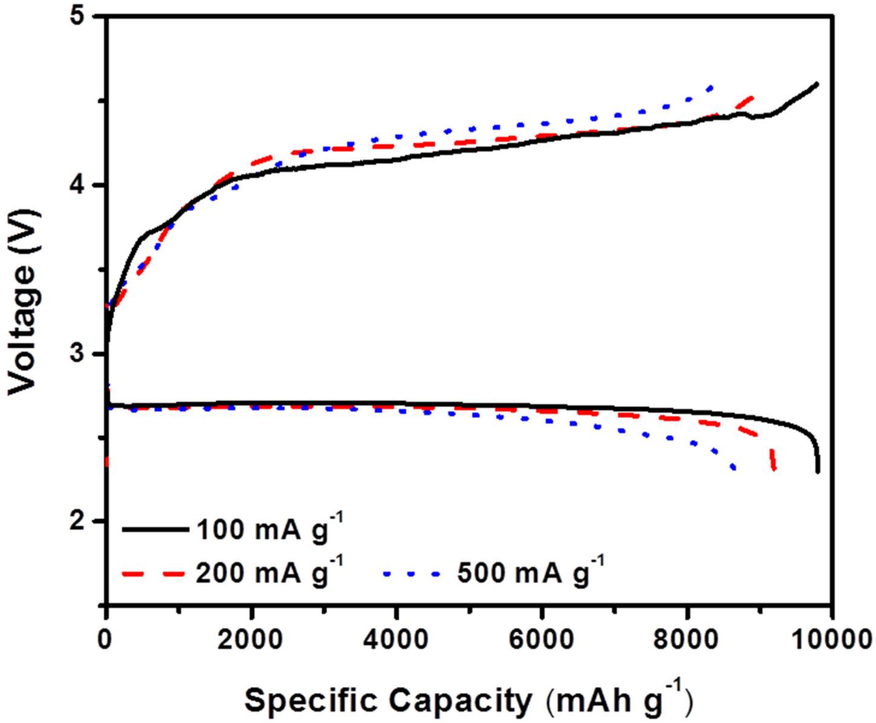

Figure S13. The rate capability of Li-O_2 batteries with PTMA electrodes with current densities from 100 to 500 mA g^{-1}. The cut-off voltage is set at 2.3 V/4.5 V.

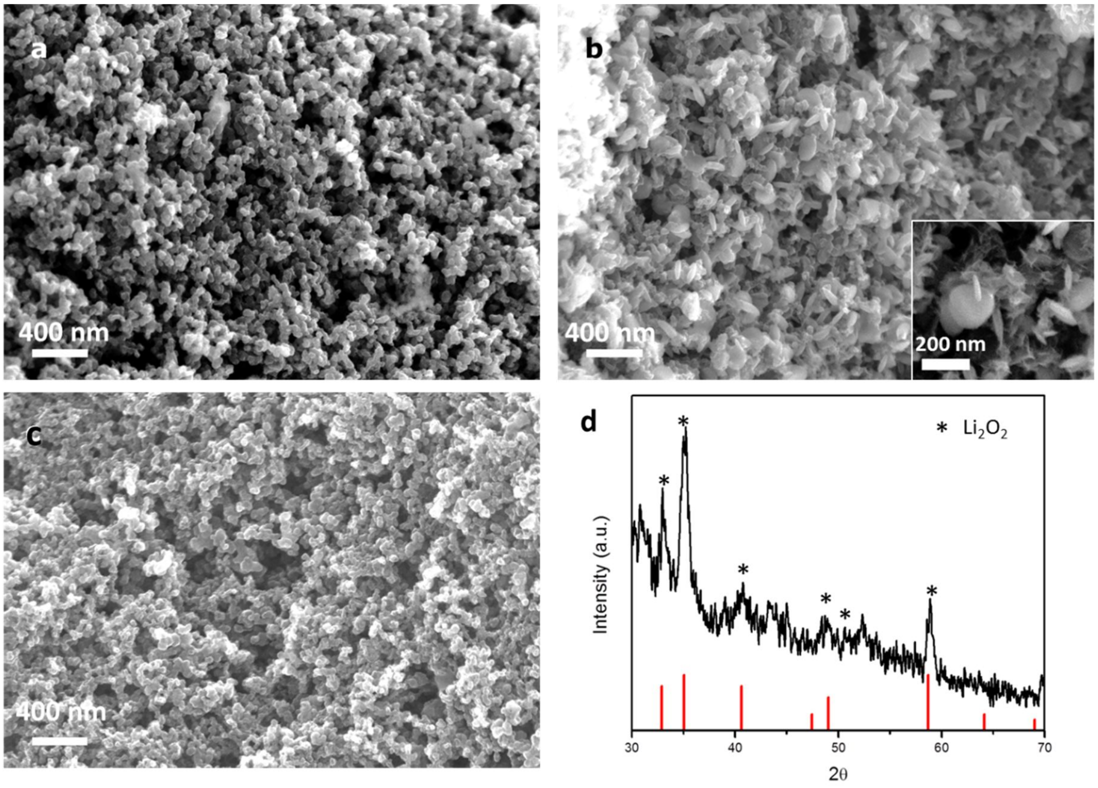

Figure S14. The SEM images of the PTMA electrode (a) before discharge, (b) after first discharge, and (c) after first charge. (d) XRD pattern of the electrode after discharge.

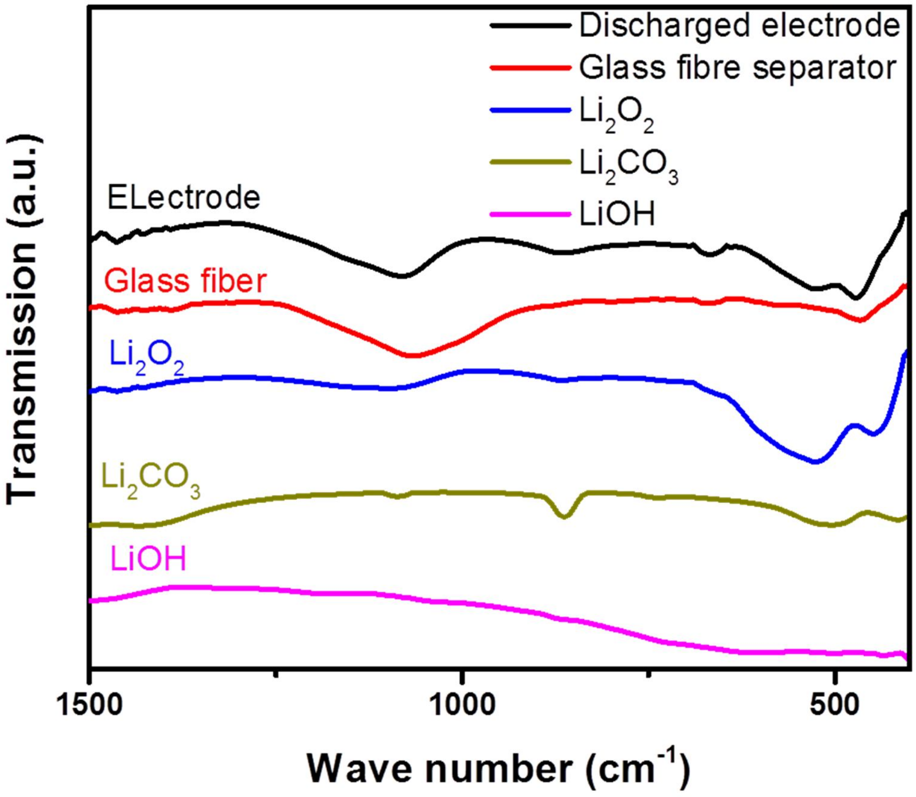

Figure S15. The FTIR spectrum of the PTMA electrode after discharge.

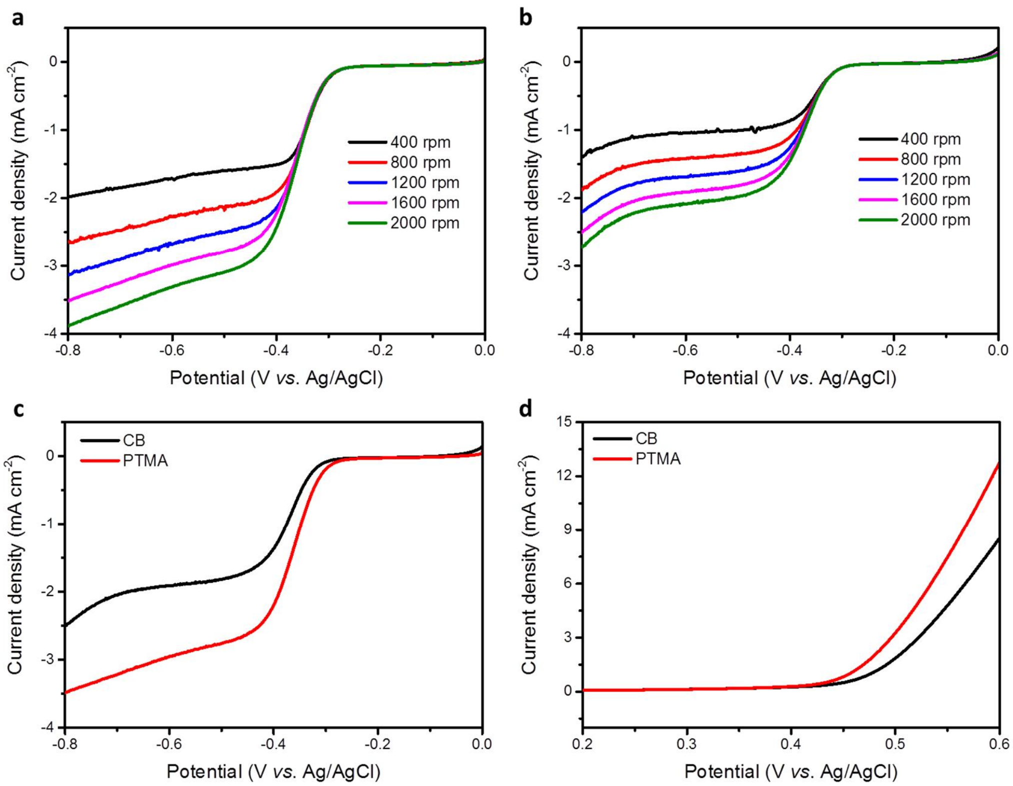

Figure S16. Aqueous ORR and OER with PTMA electrocatalyst. Operating in an O_2-saturated 1 M KOH aqueous solution, the results are electrochemically measured LSVs of (a) PTMA and (b) CB towards ORR at different rotation rates indicated; (c) ORR and (d) OER catalytic activity of PTMA and CB at a rotation rate of 1600 rpm.

To evaluate the electro-catalytic activity of PTMA, rotating disk electrode (RDE) measurements were conducted in O_2-saturated 1 M KOH aqueous solution. A comparative study was performed on CB. PTMA exhibited a pronounced electro-catalytic ORR activity associated with lower onset potential and larger current density compared to that of CB at different rotation rates in aqueous media (**a, b**). As displayed in **c**, at a potential of -0.6 (vs. Ag/AgCl), PTMA achieved a current density of 2.95 mA cm^{-2} at a rotation rate of 1600 rpm, whereas much smaller current density of 1.90 mA cm^{-2} was measured for CB. PTMA have further shown to be efficient in catalysing the oxidation of water to oxygen. According to the polarization curves (**d**), it is evident that PTMA displays better OER performance than CB, showing an over-potential of 339 mV to achieve 5 mA cm^{-2} (380 mV for CB).
